# Predicting Pre- and Post-Diagnostic Depression in Women with Abnormal Pap Screening Tests: A Neural Network Approach

**DOI:** 10.3390/life15071041

**Published:** 2025-06-30

**Authors:** Irena Ilic, Goran Babic, Sandra Sipetic Grujicic, Ivana Zivanovic Macuzic, Milena Ilic, Ana Ravic-Nikolic, Vesna Milicic

**Affiliations:** 1 Faculty of Medicine, University of Belgrade, 11000 Belgrade, Serbia; 2 Department of Gynecology and Obstetrics, Faculty of Medical Sciences, University of Kragujevac, 34000 Kragujevac, Serbia; 3 Institute of Epidemiology, Faculty of Medicine, University of Belgrade, 11000 Belgrade, Serbia; 4 Department of Anatomy, Faculty of Medical Sciences, University of Kragujevac, 34000 Kragujevac, Serbia; 5 Department of Epidemiology, Faculty of Medical Sciences, University of Kragujevac, 34000 Kragujevac, Serbia; 6 Department of Dermatovenerology, Faculty of Medical Sciences, University of Kragujevac, 34000 Kragujevac, Serbia

**Keywords:** cervical cancer screening, diagnostic procedures, depression, artificial neural networks

## Abstract

(1) Background: After receiving an abnormal Papanicolaou smear result, very often women fail to adhere to further procedures due to depression. Using a neural network approach, this research aimed to predict pre- and post-diagnostic depressive symptoms in women with abnormal Pap screening tests. (2) Methods: The study was conducted at the Clinical Center of Kragujevac, Serbia, among 172 women with a positive Pap screening result before and after diagnostic procedures (colposcopy/biopsy/endocervical curettage). Just before and 2 to 4 weeks after the diagnostic procedures, women filled out a socio-demographic questionnaire and the Hospital Anxiety and Depression Scale (HADS). Multilayer perceptron neural networks were modeled. (3) Results: Depression was present in 37.2% of women before diagnostic procedures and in 48.3% after. Feature selection showed four variables that correlated with depression before diagnostic procedures—anxiety (according to the HADS), depression according to the CESD scale, worry score on the POSM scale and use of sedatives. Model for predicting pre-diagnostic depression yielded an accuracy of 79.41%, with a value of 0.842 for area under the receiver operating characteristic curve (AUROC). The HADS anxiety score, place of residence and CESD score were the most important attributes for predicting post-diagnostic depression, with an ANN model accuracy of 88.24% and AUROC 0.939. (4) Conclusions: This research revealed a possible way of predicting depression occurrence in those women who received a positive Pap screening test and who are undergoing follow-up diagnostics, aiding medical doctors in the provision of successful and on-time psychological assistance.

## 1. Introduction

The GLOBOCAN 2022 estimates showed that cervical cancer is the fourth most frequently diagnosed cancer in women in Serbia (nearly 900 new cases) and is in fifth place among the leading causes of cancer death (nearly 500 deaths) [1]. Throughout several decades, implementation of the screening program with a Papanicolaou test resulted in a significant decrease in occurrence and deaths from cervical cancer in developed countries [2,3,4]. The implementation of organized, decentralized cervical cancer screening in Serbia began in 2013, with the basic screening test being the cytological Papanicolaou (Pap) smear of the cervix [5].

After the Pap test, Human Papillomavirus (HPV) molecular tests were designed for cervical cancer screening [6], and HPV vaccines were developed for primary prevention against cervical cancer [7,8]. HPV is identified as a necessary cause of almost all invasive cervical cancers, whereby persistent infection with high-risk types (particularly HPV 16 and 18) is considered responsible for the majority of cervical cancer cases [9]. In the cervical cancer screening process, the sensitivity of HPV testing was consistently above 96% (specificity ranged from 89 to 95%), compared with a sensitivity range of 32–88% for cytology (specificity ranged from 86% to 100%) [10,11,12]. Some identified specific molecular biomarkers associated with cervical cancer development (like HPV DNA, p16, Ki67, etc.) can provide improved accuracy and sensitivity of cervical cancer screening, predict the risk of progression to invasive cancer, predict treatment responses and enable treatment monitoring and predict survival outcomes [13]. HPV vaccines are highly effective in the primary prevention of cervical cancer, as well as in the reduction of the overall burden of HPV-related diseases [10,14,15]. Besides, primary prevention continues to play a crucial role in preventing cervical cancer, involving the promotion of healthy lifestyles and behaviors that minimize the risk of cervical cancer, such as the use of condoms for sexual intercourse, sexual partner reduction and social campaigns that increase the uptake of screening and HPV vaccination [16,17].

Yet, despite receiving an abnormal Pap result some women do not follow through with the prescribed guidelines and do not adhere to required diagnostic procedures [18,19]. Reasons for failing to adhere to follow-up following an abnormal Pap result can involve heightened depression levels [20,21]. Studies have found that the occurrence of depression following colposcopy was between 7% and 22% [22,23,24]. The majority of the available research has indicated that after colposcopy levels of depression decreased [24,25,26]. Research conducted in Great Britain [10], using the Hospital Anxiety and Depression Scale (HADS) questionnaire, found that the prevalence of depression (defined as score of ≥8 on the HADS depression subscale) was lower (6.6%) after colposcopy compared to that prior to the procedure (7.9%). One study that followed women who were referred for colposcopy following receipt of a positive Pap test within the program of organized screening, found a decrease in depression levels over two years [27,28]. The aim of our research was to build artificial neural network models that predict the occurrence of depression before and after diagnostics performed in women with an abnormal Pap screening result.

## 2. Materials and Methods

### 2.1. Setting

The study setting was at the Clinical Center of Kragujevac’s Clinic of Gynecology and Obstetrics. Following their participation in the screening program and receipt of an abnormal Pap test result, women were sent to this clinic in the period of 4–6 weeks to undergo additional diagnostics (i.e., consultative colposcopy/biopsy/endocervical curettage).

### 2.2. Study Design

This study applied a cross-sectional research design.

### 2.3. Study Sample

The sample consisted of every woman who participated in cervical cancer screening and received a positive Papanicolaou test and who followed through with diagnostics. Criteria for eligibility involved age between 20 and 65, receipt of a positive Pap smear through routine participation in population screening for cervical cancer, residing in Kragujevac and the ability to fluently speak and write in Serbian. The ineligibility criteria involved age under 20 or over 65, pregnancy during study recruitment and former cervical lesions for which treatment was administered. Women were excluded if they were undergoing treatment of reproductive organ diseases during the study, if they refused participation or if any objective reason was present and hindered study participation.

### 2.4. Sample Size Calculation

Based on the findings of Sharp and coauthors [22], frequency of depression according to the HADS among women who had an abnormal Pap screening result was 7.9% before and 16.0% following diagnostics. The statistical program Epi Info Version 7.2.0.1 (Centers for Disease Control and Prevention, Atlanta, Georgia) was used to determine the minimum necessary sample, which was calculated as 154 women, with the following parameters applied in calculation—Fleiss method with correction factor, first type error (α) 0.05 and study strength 95%.

### 2.5. Data Collection

All eligible participants completed a socio-demographic survey and the HADS. All women who took part in the study provided written informed voluntary consent before participating in this research. There were about 20 (±5) minutes at disposal for filling out the survey, and this occurred right before the diagnostics were performed (1st study time point) and after 2 to 4 weeks right before the receipt of the results of the conducted diagnostics (2nd study time point). Opting not to participate in the study was recorded. Out of 238 eligible participants, the study sample included 172 participants (participation rate: 72.3%). Questionnaires that were not fully completed are not included in the analysis.

### 2.6. Instruments

The socio-demographic survey collected data on the age of the women (≤30/31–40/41–50/51–60/≥61), residence (rural/urban), education (≤8 years/>8 years) and marital status (without partner/with partner). Additionally, HADS [29], “The Center for Epidemiologic Studies Depression” (CES-D) [30] and “Specific Process and Outcome Specific Measure” (POSM) [31] were utilized. Depressiveness is characterized as a cognitive triad that consists of negative self-perception, negative world outlook and negative future-oriented thoughts [32].

Anxiety and depression can be detected and quantified with the HADS, a self-completion instrument [29]. This tool has 14 questions corresponding to two subscales, with each subscale having 7 questions assessing anxiety and depression within a week of answering the survey. The maximum score for each subscale is 21. In this study, a lower score (0 to 7) denoted a normal level of anxiety/depression level, and a score of 8 to 21 denoted the presence of anxiety/depressive symptoms.

People who are at risk of depression can be detected using the CES-D, a self-report scale for depression screening [30]. In this research, a score ≥16 was indicative of depression. The psycho-social consequences, i.e., burden, in women who received a positive Pap screening test was assessed with the POSM, a specific questionnaire that involves two factors (i.e., worry and satisfaction with information/support) [31]. The validity and reliability of the Serbian versions of all measurement instruments used in this research were confirmed before the beginning of this research, according to the internationally accepted methodology [33,34,35].

### 2.7. Statistical Analysis

Categorical variables were assessed with methods of descriptive statistics and presented as absolute numbers and frequencies. Comparison of categorical variables was performed with the chi-squared test.

The model of artificial neural network that was built was multilayered perceptron with feedforward back-propagation. The investigated outcomes were pre-diagnostic and post-diagnostic depression. Entry variables included socio-demographic and epidemiological characteristics of participants. Attribute selection based on correlation (i.e., Pearson correlation between the attribute and the class) was used to select variables that contributed the most to the prediction model, enabling containment of the most relevant features thus reducing model complexity to help avoid overfitting. A three-way holdout was achieved by splitting the dataset into 60% for training the model, 20% for validation to tune hyperparameters and help detect overfitting and 20% for testing, i.e., external validation (separate test set considered a generalization test). A 10-fold cross-validation on the training set + validation set was used for model construction and selection of the best parameters. The built models were evaluated using the confusion matrix and kappa statistic. Model performance was assessed by estimating accuracy, false positive outcomes rate, false negative outcomes rate, precision, receiver operating characteristic (ROC) curve and Matthews correlation coefficient.

The Statistical Package for Social Sciences software (SPSS, version 20.0) from IBM, Chicago, IL, USA, was utilized to run statistical analyses, while data exploration and ANN model creation were undertaken with the Waikato Environment for Knowledge Analysis program (Weka, version 3.8.0, Waikato, New Zealand). Differences were deemed statistically significant at the *p*-value < 0.05.

### 2.8. Ethical Considerations

This study is part of a research approved by the Ethics Committee of the Faculty of Medical Sciences, University of Kragujevac (Ref. No.: 01-2176) and by the Ethics Committee of the Clinical Center Kragujevac (Ref. No.: 01-2869). All participants provided written informed voluntary consent prior to taking part in the study and confidentiality was protected.

## 3. Results

Just over half (56.4%) of the participants were aged ≤50 years (Table 1). Most of the women resided in an urban area, had a higher educational level and had a partner. Among our study participants, the frequency of symptoms of depression (according to the HADS-D subscale, with a score of 8–21) was higher after (48.3%) than before diagnostic procedures (37.2%); the difference in depression prevalence before and after diagnostics was statistically significant (*p* = 0.038). Before the diagnostic procedures, women with a positive result on the Pap screening test who had depression were mostly in the fifth and sixth decade of life (*p* = 0.006). After diagnostic procedures, women with a positive result on the Pap screening test who had depression were mostly rural residents (*p* < 0.001).

**Figure 1:**
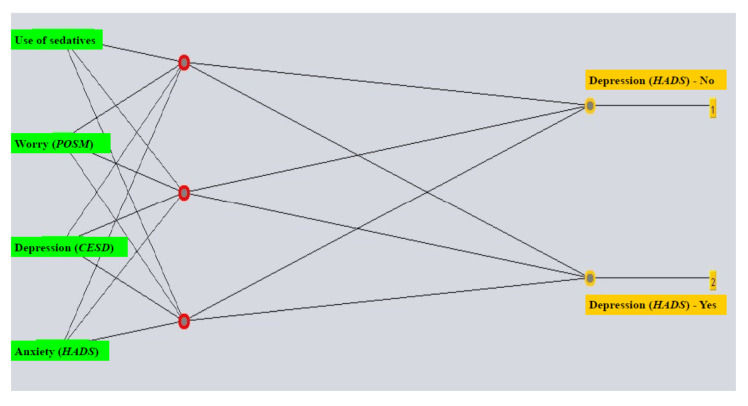
The multilayered perceptron with the most relevant attributes selected for prediction of depression prior to diagnostics. Abbreviations: HADS (Hospital Anxiety and Depression Scale); CES-D (Center for Epidemiologic Studies Depression); POSM (Process and Outcome Specific Measure).

The following parameters were used to build the ANN model for predicting depression according to the HADS-D scale before diagnostic procedures comprising all attributes: learning rate 0.3, momentum value at 0.5, training for 1000 epochs and neurons in the hidden layer equaling one half of the sum of attributes and classes. The model that included the chosen attributes used the following parameters: learning rate 0.3, momentum value at 0.5, training for 1000 epochs and three neurons in the hidden layer (Figure 1).

The choice of relevant attributes was conducted by determining the correlation of attributes with the class that is being predicted (i.e., pre-diagnostic depression), identifying four important variables: anxiety according to the HADS-A scale, depression according to the CESD scale, worry score on the POSM scale and the use of sedatives (Table 2).

**Figure 2:**
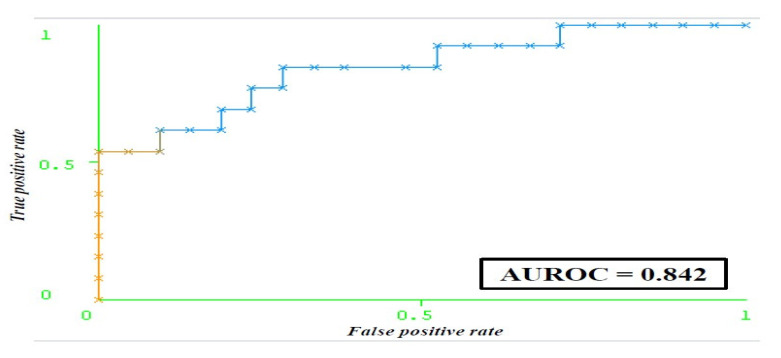
ROC curve of the artificial neural network model for predicting depression in women with a positive Pap test prior to diagnostics. Abbreviation: HADS (Hospital Anxiety and Depression Scale); AUROC (Area Under the Receiver Operating Characteristic curve).

By selecting attributes that correlate with the class (predicted outcome), models that have higher accuracy in comparison with those comprising all features were obtained. The pre-diagnostic depression prediction model showed a sensitivity of 79.4% and specificity of 69.7% (Table 3). The AUC showed that the built neural network is a very good classifier (AUC 0.842) (Figure 2).

**Figure 3:**
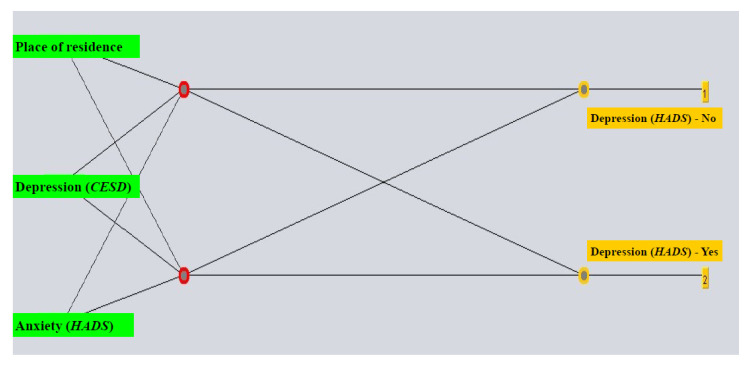
The multilayered perceptron with the most relevant attributes selected for prediction of depression following diagnostics. Abbreviations: HADS (Hospital Anxiety and Depression Scale); CES-D (Center for Epidemiologic Studies Depression).

For creating the ANN for prediction of depression following diagnostics in women with a positive Pap test, the model comprising all attributes employed the following parameters: learning rate 0.3, momentum value at 0.2, training for 1000 epochs and two hidden layers, for which the number of neurons in one equaled one half of the sum of the number of attributes and number of classes and the other the sum of the number of attributes and number of classes. The ANN with chosen attributes employed a learning rate of 0.4, a momentum value at 0.2, training for 1000 epochs and number of neurons in the hidden layer equaling half of the sum of the number of attributes and classes (Figure 3).

As the most important features for predicting post-diagnostic depression, the following were selected: anxiety according to the HADS-A scale, place of residence and CESD score (Table 2). The post-diagnostic depression prediction model had the highest sensitivity and specificity (88.2% and 89.6%, respectively) (Table 4).

**Figure 4:**
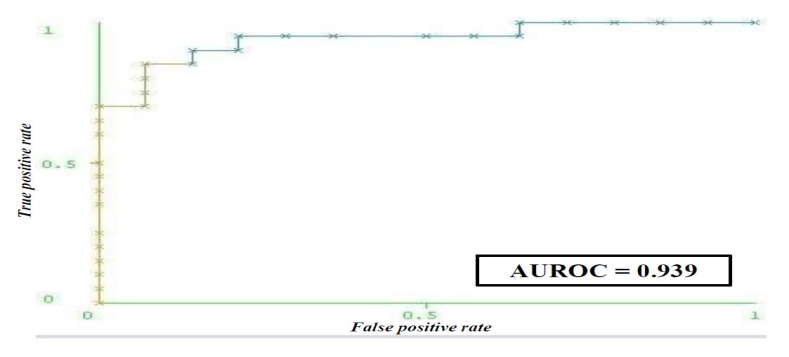
ROC curve of the artificial neural network model for predicting depression in women with a positive Pap test following diagnostics. Abbreviation: HADS (Hospital Anxiety and Depression Scale); AUROC (Area Under the Receiver Operating Characteristic curve).

The probability that a woman for whom the model predicted that she does not have depression actually does not have depression is 94.4%. Excellent model performance for prediction is reflected in a very high agreement between the true and predicted class (*MCC* = 0.768) as well as in the AUROC (Figure 4).

## 4. Discussion

This research indicated that for women who had an abnormal Pap screening result, the features that help predict pre-diagnostic depression according to the HADS scale were anxiety according to the HADS-A scale, depression according to the CESD scale, worry score on the POSM scale and sedatives use, while for predicting post-diagnostic depression, the selected correlates included anxiety according to the HADS-A scale, place of residence and CESD score.

Information about the experiences of women who are undergoing colposcopy and other diagnostic procedures in Serbia are limited. According to the available literature, there are no results of similar studies, and no research was undertaken in women with a positive Pap screening test regarding the level of depression prior to and after diagnostic procedures, nor were there studies on the application of ANNs with the aim of predicting pre- and post-diagnostic depression in this population. So far, the research has involved the development of ANN models for predicting depression in high school students in China [36], depression in the Indian geriatric population [37] and post-partum depression among pregnant women in Spain [38]. A study in America [39] found the accuracy of predicting depression following a concussion to be 76.96%, which is in line with the accuracy obtained in our research.

Our research confirmed what previous evidence has shown, namely that significant correlates of depression in women with an abnormal Pap smear before and after diagnostics were a higher level of anxiety and a higher level of POSM-worry [40]. Receiving an abnormal Pap test and undergoing additional diagnostic procedures sometimes leads to heightened levels of stress, the experience of pain throughout diagnostics and therapeutic interventions, concerns about future offspring, experiencing self-blame and perceived inadequacy of external support [41]. Increased distress is probably due to some other psychological and social issues that women are experiencing, such as health-related concerns, worry about sexual functioning, fear about future fertility, fear of developing cervical cancer or body image [42,43,44]. Following colposcopy, significantly more common were low scores on the mental health component of quality of life and notable anxiety [45]. Also, dimensions of sleep and sexual activity had significantly lower values in women with positive cervical cytology based on colposcopy compared to the general population [46].

To date, research has documented sedative use throughout screening for cervical cancer and having a positive Pap smear [47] and also in women who received a cervical cancer diagnosis [48]. In addition, findings of a Swedish cohort study suggest that patients with cervical cancer who have a preexisting diagnosis of a mental disorder (e.g., depression, anxiety, as well as substance abuse, etc.) have worse survival, whereby a higher risk of death was recorded for those with substance abuse (such as sedatives or hypnotics, cocaine and other psychoactive substances) [49]. The correlation of the use of sedatives with depression after diagnostic procedures found in our study can include the fear of cancer and/or fear of the unknown while waiting to receive results of additional examinations, unsatisfactory support from the environment and insufficient information regarding the meaning of an abnormal Pap test, which may also be a consequence of the organized screening program for cervical cancer being only recently introduced in Serbia.

In this study, an urban place of residence was a feature chosen as a significant correlate of post-diagnostic depression. This could be related to the fact that, on average, women who live in the city have higher levels of education and income; an increased level of awareness, knowledge and preventive health behaviors and also have health insurance (they often have private insurance as well) [50,51]. In addition, an urban place of residence is characterized by a greater availability of medical services, access to health care practitioners and, consequently, better information about screening for cervical cancer, the Pap test result, meaning of the term dysplasia and additional diagnostic procedures. Also, community factors include patient concerns about the confidentiality of results (especially in rural areas), lack of awareness of the importance of follow-up and cultural beliefs [52]. However, when interpreting the correlation with an urban place of residence, it should be considered that urban areas may overlap with rural ones and that, consequently, misclassifications may occur, which could at least partially obscure the observed differences in depression levels in this research. Nevertheless, these findings underline the necessity of dedicated persistent efforts in providing cervical cancer screening to women who reside in rural areas of Serbia.

Despite decades of practice of mass screening for cervical cancer in most developed countries, it is still not fully known what the frequency of pre-diagnostic and post-diagnostic depression is and also what are the predictors of this effect of diagnostic procedures. The existence of depressive symptoms prior to diagnostics and persistence following diagnostics highlight the importance of identifying this subgroup of women with an abnormal Pap smear undergoing further diagnostics to help address and reduce psychological distress through the provision of appropriate information and counseling. This research developed a model that enables prediction of depression prior to and following diagnostics in women who have an abnormal Pap screening test. Some of our results of the performance of built artificial neural network models, which are characterized by higher sensitivity and specificity, could, in the context of the importance of the issue of the quality of cervical cancer screening programs, be of importance in order to improve performance characteristics of screening for cervical cancer.

In cervical cancer research, artificial neural networks have been used to classify cytological findings [53,54,55] and predict survival [56]. One of the most common applications of artificial neural networks in medicine is in the field of cervical cytopathology: the PAPNET system (Papernet System, Cytologic Screening System for Quality Control of Cervical Smears; Neuromedical Systems, Inc., Suffern, NY), an automated system for cytological screening of cervical smears, shows better or equal accuracy compared to light microscopy [54,57]. A large meta-analysis which included 77 studies showed the significance of AI in improving prevention and early diagnosis of cervical cancer [58]. A recent review of the application of AI in mental healthcare showed its use to still be developing in cancer research [59]. Reports of research conducted in breast cancer patients noted that created classifiers showed an accuracy between 74% and 83% for assessing patients for poor overall mental health (including depression) and quality of life and predicting well-being [60] and an accuracy around 74–79% for predicting deterioration in HADS scores [61]. Thus, our and the available research indicate the possibility of employing personalized risk assessment that incorporates AI techniques at the level of primary healthcare as well as in the specialist practice, that could help identify those persons who require additional support and psychological interventions, which could in turn improve both the psychological health outcomes as well as the diagnostic and treatment adherence and outcomes of the patients. In summary, using artificial neural networks presents a potential force in health care, with the potential to enhance patient care, disease prevention, reduce errors, broaden medical knowledge, improve survival and reduce health care costs. This study showed that artificial neural network models could be integrated into primary health care and gynecologic practice as screening tools for psychological risk.

### Strengths and Limitations of the Research

According to the available worldwide literature, there does not seem to be much research which has investigated depressive symptoms before and after diagnostics among women who received an abnormal Papanicolaou screening test. According to the existing research, until now there were no studies dedicated to the application of artificial neural networks and the creation of predictive models for pre- and post-diagnostic depression in women with a positive Papanicolaou screening smear. Also, another strength of the present study is that only questionnaires that are validated were used [32,33,34]. Nevertheless, this research has several limitations. The limitations of this study involve intrinsic drawbacks of the cross-sectional design, the use of self-reported surveys, the risk of information bias, the study sample size, the representativeness of the study sample, no assessment of depression level prior to the Pap smear, a lack of clinical confirmation of depression throughout the study, a lack of insight into medical documentation of study participants, the absence of data on some other characteristics of research respondents (i.e., HPV status, socio-economic status, comorbidities, etc.) as well as the inability to dismiss the effect of possible exposure to some other factors on levels of depression pre- and post-diagnostics.

## 5. Conclusions

Women who receive a positive Pap result, both prior to and following additional diagnostics, have a considerable level of depressive symptoms. The developed ANNs make it possible to identify women with an abnormal Pap screening smear test who are at risk of depression prior to and after diagnostic procedures. These findings could enable better support to women during all procedures of the organized cervical cancer screening program and improved coverage with medical procedures and could significantly contribute to improved survival.

## Figures and Tables

**Table 1:** Women with a positive Pap test (N = 172): socio-demograpfic features and frequency of depression prior to and following diagnostics.

		Depression—Current			
Variables	Total	Prior to Diagnostics		Following Diagnostics	
	Number (%)	Number (%)	*p* *	Number (%)	*p* *
Age (years)					
- ≤30	12 (7.0)	2 (3.1)		5 (6.0)	
- 31–40	43 (25.0)	9 (14.1)		17 (20.5)	
- 41–50	42 (24.4)	20 (31.2)		24 (28.9)	
- 51–60	51 (29.7)	21 (32.8)		30 (36.1)	
- ≥61	24 (14.0)	12 (18.8)	0.006	7 (8.4)	0.789
Place of residence					
- Rural	45 (26.2)	22 (34.4)		38 (45.8)	
- Urban	127 (73.8)	42 (65.6)	0.084	45 (54.2)	<0.001
Education level					
- ≤8 years	37 (21.5)	17 (16.6)		18 (21.7)	
- >8 years	135 (78.5)	47 (73.4)	0.216	65 (78.3)	0.957
Marital status					
- Without partner	33 (19.2)	13 (20.3)		19 (21.3)	
- With partner	139 (80.8)	51 (79.7)	0.773	70 (78.7)	0.457
Depression (HADS-D score ≥ 8)					
- No		108 (62.8)		89 (51.7)	
- Yes		64 (37.2)		83 (48.3)	0.038

Abbreviation: HADS-D (Hospital Anxiety and Depression Scale—Depression subscale). *p* (probability: * χ^2^-test).

**Table 2:** Selection of features for predicting depression (according to HADS) based on correlation for identification of relevant prediction attributes in women with a positive Pap test prior to and following diagnostics.

Attributes	Depression—Prior	Depression—Following
	Pearson Correlation Coefficient	
HADS score for anxiety	0.62003	0.73337
CESD score for depression	0.41789	0.32923
POSM score for worry	0.29043	
Use of sedatives	0.24591	
Place of residence		0.41539

Abbreviations: HADS (Hospital Anxiety and Depression Scale); CES-D (Center for Epidemiologic Studies Depression); POSM (Process and Outcome Specific Measure).

**Table 3:** Performance metrics of the built artificial neural network for prediction ofdepression in women with a positive Pap test prior to diagnostics (according to HADS).

Evaluation Metrics of the Model	Training Set + Validation ** Including All Attributes	Test Set Including All Attributes	Training Set + Validation ** Including Chosen Attributes	Test Set Including Chosen Attributes
Accuracy	73.913%	70.588%	84.782%	79.411%
Kappa	0.430	0.358	0.684	0.529
TP Rate *	0.739	0.706	0.848	0.794
FP Rate *	0.315	0.358	0.138	0.303
Precision * (PPV)	0.736	0.700	0.859	0.810
NPV	0.659	0.636	0.750	0.875
ROC Area *	0.793	0.678	0.889	0.842
MCC	0.432	0.361	0.691	0.562

Abbreviations: HADS (Hospital Anxiety and Depression Scale); TP (True Positive rate); FP (False Positive rate); PPV (Positive Predictive Value); NPV (Negative Predictive Value); ROC (Receiver Operating Characteristic curve); MCC (Matthews correlation coefficient). * pondered arithmetic mean for both classes; ** 10-fold cross validation.

**Table 4:** Performance metrics of the built artificial neural network for prediction of depression in women with a positive Pap test following diagnostics (according to HADS).

Evaluation Metrics of the Model	Training Set + Validation ** Including All Attributes	Test Set Including All Attributes	Training Set + Validation ** Including Chosen Attributes	Test Set Including Chosen Attributes
Accuracy	77.536%	61.764%	89.855%	88.235%
Kappa	0.550	0.250	0.797	0.762
TP Rate *	0.775	0.618	0.899	0.882
FP Rate *	0.222	0.353	0.095	0.104
Precision * (PPV)	0.778	0.648	0.902	0.890
NPV	0.735	0.733	0.855	0.944
ROC Area *	0.842	0.725	0.924	0.939
MCC	0.552	0.262	0.800	0.768

Abbreviations: HADS (Hospital Anxiety and Depression Scale); TP (True Positive rate); FP (False Positive rate); PPV (Positive Predictive Value); NPV (Negative Predictive Value); ROC (Receiver Operating Characteristic curve); MCC (Matthews correlation coefficient). * pondered arithmetic mean for both classes; ** 10-fold cross validation.

## Data Availability

Data is contained within the article.
